# Transarterial strategies for the treatment of unresectable hepatocellular carcinoma: A systematic review

**DOI:** 10.1371/journal.pone.0227475

**Published:** 2020-02-19

**Authors:** Biao Yang, Jie Liang, ZiYu Qu, FangYun Yang, ZhengYin Liao, HongFeng Gou

**Affiliations:** 1 Department of Gastroenterology, West China Hospital, West China Medical School, Sichuan University, Chengdu, P.R. China; 2 School of Public Health, Chengdu University of Traditional Chinese Medicine, Chengdu, P.R. China; 3 Department of Head and Neck Oncology, Cancer Center and State Key Laboratory of Biotherapy, West China Hospital, West China Medical School, Sichuan University, Chengdu, P.R. China; Texas A&M University, UNITED STATES

## Abstract

Conventional transarterial chemoembolization (cTACE), drug-eluting beads (DEB-TACE) and transarterial radioembolization (TARE) are alternative strategies for unresectable hepatocellular carcinoma (HCC). However, which of these strategies is the best is still controversial. This meta-analysis was performed to evaluate the effects of DEB-TACE, TARE and cTACE in terms of overall survival (OS), tumor response and complications. A literature search was conducted using the EMBASE, PubMed, Google Scholar, and Cochrane databases from inception until July 2019 with no language restrictions. The primary outcome was overall survival, and the secondary outcomes included complete response and local recurrence. The comparison of DEB-TACE with cTACE indicated that DEB-TACE has a better OS at 1 year (RR 0.79, 95% CI 0.67–0.93, p = 0.006), 2 years (RR 0.89; 95% CI 0.81–0.99, p = 0.046), and 3 years (RR 0.89; 95% CI 0.81–0.99, p = 0.035). The comparison of TARE with cTACE indicated that TARE has a better OS than cTACE at 2 years (RR 0.87; 95% CI 0.80–0.95, p = 0.003) and 3 years (RR 0.90; 95% CI 0.85–0.96, p = 0.001). The comparison of DEB-TACE with TARE indicated that DEB-TACE has a better OS than TARE at 2 years (RR 0.40; 95% CI 0.19–0.84, p = 0.016). The current meta-analysis suggests that DEB-TACE is superior to both TARE and cTACE in terms of OS. TARE has significantly lower complications than both DEB-TACE and cTACE for patients with HCC. Further multicenter, well-designed randomized controlled trials are needed, especially for evaluating DEB-TACE versus TARE.

## Introduction

Hepatocellular carcinoma (HCC) is the fifth most common cancer[1, 2]. Treatments of HCC is widely guided by Barcelona Clinic Liver Cancer (BCLC) staging system[2]. For intermediate HCC, conventional transarterial chemoembolization (cTACE) has been recommended as the standard therapy[2]. cTACE is based on injection of chemotherapeutic agents and selective vascular embolization into the arteries feeding the tumor[3], resulting in a high intratumoral concentration of chemotherapeutic agents as well as strong cytotoxic effects[4].

In recent years, both drug-eluting beads (DEB-TACE) and transarterial radioembolization (TARE) have been considered as alternative therapies to cTACE for unresectable HCC. DEB-TACE involves the selective application of chemotherapy-loaded microbeads which embolize the tumor arteries and ensure the loaded chemotherapeutic agent slowly releases to achieve a lower systemic drug peak compared to cTACE [5, 6]. Song et al[7] showed that the overall survival rates at 6, 12, and 18 months were 93%, 88%, and 88%, respectively, in the DEB-TACE group, which were better than those in the cTACE group (80%, 67%, and 61%, respectively). These results are similar to those obtained in three other studies [8–10]. However, a recent RCT performed by Golfieri et al[11] showed that DEB-TACE and cTACE were equally effective regarding 1- and 2-year survival rates(DEB-TACE vs. cTACE; 86.2%vs. 86.2%; 56.8% vs. 56.8%) (p = 0.95).

TARE, using resin microspheres or a glass matrix labeled with yttrium-90, is another regional technique. TARE, which consists of the arterial infusion of microspheres integrated to a radiotherapeutic agent, allows for the concentration of beta-radiation in the tumor parenchyma without damaging the surrounding liver tissue [12, 13]. It seems to be tumor-selective based on natural disruptions to the microvasculature surrounding liver tumors [14] and can be selectively delivered with whole, lobar or segmental-liver approaches [15]. Soydal et al[16] reported that the mean OS was significant longer with TARE than with cTACE (39.24±4.62 vs. 30.63 ± 3.68, respectively, p = 0.014). The respective 1- and 2-year survival rates were higher for TARE (72%, 74%) than for cTACE (47%, 59%)[16]. These findings were confirmed by Lewandowski et al[17]. However, Kolligs et al[18] found that 46.2% and 66.7% of patients in the TARE and cTACE study arms were alive at 12 months.

Only a few studies have compared DEB-TACE and TARE. Akinwande et al[19] showed that OS was higher with DEB-TACE than TARE (15 vs. 6 months, respectively, p<0.0001). This finding is inconsistent with the results of Lance et al[20], who demonstrated there was no significant difference in the median OS between radioembolization and chemoembolization (8.0 vs. 10.3 months, respectively, p = 0.33). McDevitt et al[21] found no significant difference in the median overall survival between DEB-TACE and TARE after treatment (9.9 vs. 8.1 months, respectively, p = 0.11).

Based on these studies, the best transarterial strategy for unresectable HCC (cTACE, DEB -TACE, and TARE) is unclear. Hence, the purpose of this meta-analysis was to systematically analyze the published data comparing DEB-TACE, TARE and cTACE for the treatment of unresectable HCC in terms of the OS, tumor response rate and complications.

## Methods

### Literature search

This study was approved by the Local Ethics Committee of West China Hospital, Sichuan University. This meta-analysis strictly followed the Preferred Reporting Items for Systematic reviews and Meta-Analyses (PRISMA) guidelines (S1 File) [22]. A comprehensive search of the PubMed, EMBASE, Google Scholar, and Cochrane databases from inception until July 2019 with no language restrictions was performed. Search terms included the medical subject headings “chemoembolization, therapeutic” and “liver neoplasms”, and the free text words “transarterial chemoembolization”, “radioembolisation”, “TheraSphere”, “SIR-spheres”, “yttrium-90”,” drug-eluting beads”, “DC bead”, “QuadraSphere”, “CalliSpheres” and “HepaSphere”(S2 File).

### Inclusion and exclusion criteria

Clinical studies were required to fulfill the following inclusion criteria: 1) study design: randomized controlled trials, retrospective or prospective cohort studies; 2) population: patients with HCC confirmed by typical imaging scans or pathology; 3) interventions: DEB-TACE directly compared to cTACE, or TARE compared with cTACE, or DEB-TACE compared with TARE; and 4) outcomes: studies included efficacy and/or complications. The exclusion criteria were as follows: 1) abstracts, letters, systematic reviews, case series or studies lacking control groups; 2) the outcomes of interest were not reported; 3) studies with potential bias or data cannot be exacted; and 4) studies in patients with multiple malignancies.

#### Study selection

The quality of the included studies was independently assessed by three reviewers (authors 1 to 3). The three reviewers independently read both the titles and abstracts to assess the eligible studies. The full texts of the potential studies were carefully examined for inclusion. Any disagreements were addressed by discussion.

### Data extraction

Two reviewers (author 1 and author 2) independently extracted the following data from each study: basic study information (author, publication year, study design, and region), patient characteristics (age, sex, BCLC stage, tumor number, tumor size, AFP levels, Child-Pugh class, MELD score, and ECOG score), and clinical outcomes (complications, OS, and tumor response,). The mean and standard deviation (SD) was extracted. Most of the original data was extracted directly from the studies, while part of the data in terms of OS and tumor response was extracted via curves using the software Engauge Digitizer (version 4.1) provided by Parmar [23] and analyzed by using a Microsoft Excel spreadsheet described by Tierney et al[24].

### Endpoints

The primary outcome was OS, and the secondary outcome were both tumor response and complications.

### Quality assessment of the selected studies

The quality of the included nonrandomized studies was assessed by using the modified Newcastle-Ottawa scale which ranged from 0 to 9 points, and studies with ≥ 8 points were considered high quality[25]. The quality items assessed included early stopping, sequence generation, blinding, allocation concealment, incomplete outcome data, baseline balance, and selective outcome reporting. The randomized controlled trials were assessed by using the Jadad score according to the study design, risk of bias, and inconsistency, indirectness, and imprecision of the results [26]. Studies with ≥4 points were considered high quality. The publication bias for the primary endpoint were assessed by using Begg’s and Egger’s tests. Any disagreements of the quality assessment were arbitrated by a third reviewer (author 3).

### Statistical analysis

Statistical analyses were conducted with using Stata 12 (Stata Corporation, College Station, TX, USA). Risk ratios (RR) with 95% confidence intervals (CIs) were calculated for OS at 1, 2, and 3 years. Hazard ratios (HR) with 95% CIs were calculated for factors related to survival. Odds ratios (OR) with 95% CIs were calculated for the incidence of tumor response and complications. Heterogeneity was assessed by Cochrane Q statistics and the I^2^ test[27]. A meta-regression analysis was conducted with covariates including study design, baseline proportion of HBV/HCV infection, BCLC stage, Child-Pugh classification and treatment sessions to evaluate the heterogeneity across studies. The significantly heterogeneous studies were excluded. A fix-effects model was used to pool the studies without significant heterogeneity, as determined by the the inconsistency index (I^2^ ≤ 50%) and chi-squared test (p > 0.05). The significance of the pooled estimates was determined by the Z-test. The p value of <0.05 was considered as statistical significance[27].

## Results

### Identification of eligible studies

**Fig 1:**
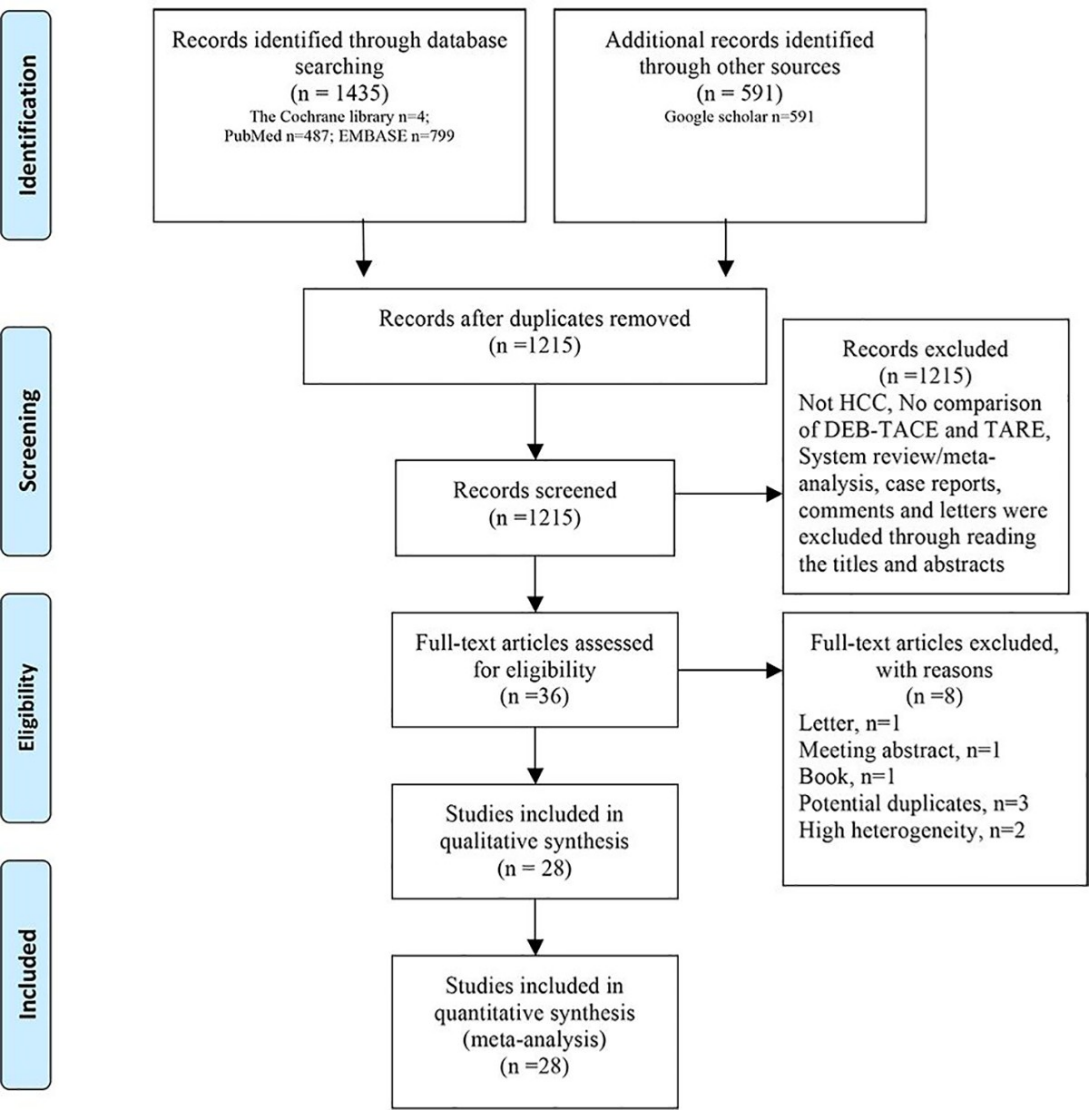
Flow chart depicting the study selection process.

A comprehensive search strategy identified 2,026 potential citations. After excluding the duplicates, there were 1215 references. An additional 1177 studies were excluded after reading the titles and abstracts. The full texts of the remaining 36 studies were carefully read. Eight studies were further excluded due to the following reasons: publication as a letter (1 study), meeting abstract (1 study), or book (1 study); potential duplicates (3 studies); and high heterogeneity (2 studies). Finally, 17 retrospective studies [7–9, 14, 16, 17, 20, 21, 28–36], three prospective studies[10, 19, 37] and eight randomized controlled trials [11, 18, 38–43] were eligible based on the inclusion criteria (Fig 1). Of these included studies, four studies compared DEB-TACE with TARE[19–21, 39], eight compared TARE with cTACE [14, 16–18, 29, 34, 37, 41], and 14 compared DEB-TACE with cTACE [7–9, 11, 28, 30–33, 36, 38, 40, 42, 43]. A total of 3438 patients were included in the studies. Of these patients, 121 patients assigned to the DEB-TACE group were compared with 122 patients in the TARE group, 351 patients assigned to the TARE group were compared with 951 patients in the cTACE group, and 862 patients assigned to the DEB-TACE group were compared with 1031 patients in the cTACE group (S1 Table).

### Characteristics of the eligible studies

The mean patient age ranged from 61 to 71.8 in the DEB-TACE vs. TARE comparison, from 58.3 to 68 years in the TARE vs. cTACE comparison, and from 55.6 to 71.3 years in the DEB-TACE vs. cTACE comparison. The average number of treatment sessions ranged from 1.37 to 3.8 in the DEB-TACE vs. TARE comparison, 1.0 to 3.4 in the TARE vs. cTACE comparison, and 1.1 to 4.0 in the DEB-TACE vs. cTACE comparison. The mean tumor size ranged from 6.0 to 9.0 cm in the DEB-TACE vs. TARE comparison, from 2.6 to 7.4 cm in the TARE vs. cTACE comparison, and from 1.8 to 8.89 cm in the DEB-TACE vs. cTACE comparison. The liver function of the included patients with Child-Pugh classifications of A/B/C were similar [(DEB-TACE vs. TARE, n = 68/48/5 vs. n = 68/49/4), (TARE vs. cTACE, n = 151/89/12 vs. n = 154/83/14), and (DEB-TACE vs. cTACE, n = 1/17/20 vs. n = 0/18/20)]. The BCLC stages of the included patients were similar [(DEB-TACE vs. TARE, n = 35/123/26/35 vs. n = 33/127/21/35), (TARE vs. cTACE, n = 180/388/69/184 vs. n = 426/426/116/434) and (DEB-TACE vs. cTACE, n = 565/163/15 vs. n = 641/192/19)]. The numbers of patients with a history of HBV/HCV/Alcohol/others were as follows: DEB-TACE vs. TARE (6/29/16/33 and 0/38/31/28), TARE vs. cTACE (20/82/72/68 vs. 105/226/241/247), and DEB-TACE vs. cTACE (213/180/153/141 vs. 249/179/247/130). The patient characteristics are summarized in Table 1, and the details of the study endpoints are summarized in S2 Table.

**Table 1:** Patient characteristics of each study.

First Author	Treatment	Number(n)	Age* (year)	Gender (M/F,n)	HBV/HCV/Alcohol /others (n)	ECOG (0/1/2/3,n)	Child-Pugh (A/B/C,n)	BCLC stage (A/B/C/D,n)	Treatment sessions* (n)	Biggest Tumor size* (cm)	Lesion location (Unilobar/ Bilobar,n)	Liver involvement (≤50%/>50%,n)	PVTT (yes/no,n)	Total Bilirubin (mg/dl,n)	ALT (U/L,n)	AFP(ng/ml)
Pitton 2015[39]	DEB-TACE	12	70.5±9.0	10/2	1/4/5/3	NA	9/3/0	1/11/0/0	3.8±2.6	6.1±3.76	5/7	12/0	NA	164±529	NA	164±529
	TARE	12	71.8±7.2	8/4	0/5/5/2	NA	10/2/0	0/12/0/0	1.5±0.5	6.1±3.64	4/8	12/0	NA	3308±10204	NA	3308±10204
McDevitt 2017	DEB-TACE	26	64 (51–85)^#^	22/4	1/5/4/16	8/10/7/1	18/8/0	0/6/20/0	1.5±1.1	8.9	-/19	23/3	6/20	> 400/≤400 13/13	NA	> 400/≤400 13/13
[21]	TARE	24	61 (53–86)^#^	21/3	0/9/3/12	7/10/6/1	15/9/0	0/5/19/0	1.6±0.5	9.0	-/17	22/2	5/14	> 400/≤400 7/17	NA	> 400/≤400 7/17
Akinwande 2016[19]	DEB-TACE	48	61.5 (19–81)^#^	40/8	4/9/4/0	<1/≥1 36/12	17/26/5	NA	2	NA	NA	47/1	14/20	43.70 (2–37424)	>250/≤250 0/48	43.70 (2–37424)#
	TARE	48	66.5 (27–82)^#^	33/8	0/11/15/0	<1/≥1 3/45	16/28/4	NA	1.67	NA	NA	45/3	16/16	26.5 (2–61378)	>250/≤250 0/48	26.5 (2–61378)#
Lance 2011[20]	DEB-TACE	35	61 (51–84)^#^	28/7	0/11/3/14	6/18/10/2	24/11/0	NA	1.5±0.13	6.0	-/26	35/0	NA	≥400/<400 16/19	NA	≥400/<400 16/19
	TARE	38	63 (44–85)^#^	33/5	0/13/8/14	6/24/7/1	31/7/0	NA	1.37±0.1	6.1	-/22	38/0	NA	≥400/<400 14/24	NA	≥400/<400 14/24
Carr 2010[29]	TARE	99	NA	70/29	9/30/37/-	NA	NA	NA	NA	NA	-/43	NA	28/71	3–1.5/<1.5 13/86	NA	≥25/<25 58/41
	cTACE	691	NA	518/173	97/132/217/-	NA	NA	NA	NA	NA	-/354	NA	295/396	3–1.5/<1.5 173 /518	NA	≥25/<25 465/226
Kooby 2010[14]	TARE	27	58.7±10.8	23/4	-/10/-/-	NA	13/14/0	NA	1.2±1.1	7.4±3.2	NA	NA	14/13	NA	NA	801±1037
	cTACE	44	61.0±9.9	36/8	-/25/-/-	NA	22/22/0	NA	1.3±1.2	7.4±5.1	NA	NA	13/31	NA	NA	690±976
Lewandowski 2009[17]	TARE	43	68	38/5	2/14/9/15	NA	24/19/0	0/34/9/0	1.8(1–6)	5.6	NA	NA	0/43	>2/≤2 6/37	NA	NA
	cTACE	43	65	36/7	6/16/10/7	NA	23/18/2	0/37/4/2	2.0 (1–5)	5.7	NA	NA	0/43	>2/≤2 10/33	NA	NA
Moreno 2013[34]	TARE	61	64	49/12	0/8/12/41	51/7/3/0	53/8/0	12/34/14/0	NA	5.0	NA	NA	NA	0.80 (0.63–1.48)	51 (19–249)	22.1 (1.9–397000)
	cTACE	55	66	43/12	0/7/13/35	40/15/0/0	44/11/0	23/13/19/0	NA	5.0	NA	NA	NA	1.1 (0.80, 1.60)	54 (23–890)	52 (0.6–264300)
Salem 2016[41]	TARE	24	62 (58–65)	17/7	3/12/4/3	NA	12/12/0	18/6/0/0	1.3±0.5	3.0	17/7	NA	0/24	1.3 (1.2–1.7)	NA	<200/≥200 21/3
	cTACE	21	64 (62–70)	16/5	1/10/1/5	NA	15/6/0	17/4/0/0	1.7±1.1	2.6	14/7	NA	0/21	0.9 (0.8–1.5)	NA	<200/≥200 19/2
El 2015[37]	TARE	44	66.1±8.9	36/8	6/8/10/9	44/0/0/0	37/7/0	0/44/0/0	1.4±0.6	6.4 (2.2–21)	24/20	44/0	NA	>2/≤2 5/39	NA	11 299±46452
	cTACE	42	58.3±6.7	38/4	1/36/0/0	42/0/0/0	33/9/0	0/42/0/0	2.2±1.4	5.7 (2.5–15)	40/2	42/0	NA	>2/≤2 3/39	NA	697.6±2834
Kolligs 2015[18]	TARE	13	65.8±6.73	11/2	NA	10/3/0/0	12/1/0	5/5/3/0	1±0.0	NA	6/7	NA	NA	Median 1.00	NA	636.0±2171.8
	cTACE	15	66.7±9.04	13/2	NA	12/3/0/0	13/2/0	4/8/3/0	3.4±2.90	NA	11/4	NA	NA	Median 1.08	NA	2624.7±9525.3
Soydal 2016[16]	TARE	40	62.28±9.73	33/7	NA	NA	0/28/12	NA	1±0	6.55±4.60	NA	38/2	NA	0.94±0.48	52.71±39.04	NA
	cTACE	40	66.15±7.81	34/6	NA	NA	0/34/6	NA	2.8±1.1	7.12±3.85	NA	35/5	NA	1.04±0.90	47.79±28.18	NA
Arabi 2014[28]	DEB-TACE	35	67.1±9.6	24/11	10/19/-/-	NA	24/11/0	NA	1.45	6 (1.3–16.3)	NA	NA	NA	median 5.5	median 74	NA
	cTACE	19	66.7±9.6	15/4	7/8/-/-	NA	17/2/0	NA	1.31	7(3–12.3)	NA	NA	NA	median 3	median 7	NA
Dhanasekaran 2010[30]	DEB-TACE	45	59.96±11.45	35/10	5/20/7/13	NA	22/11/12	NA	1.27±0.6	5.49±4.29	NA	NA	11/34	NA	NA	NA
	cTACE	26	58.96±13.3	19/7	3/11/3/9	NA	11/11/4	NA	1.46±0.8	7.40±4.91	NA	NA	2/24	NA	NA	NA
Kloeckner 2015[31]	DEB-TACE	76	NA	68/8	10/20/30/10	53/20/2/1	51/22/3	8/34/30/4	2.96±1.79	NA	NA	NA	11/65	≥2/<2 12/64	NA	NA
	cTACE	174	NA	140/30	14/47/86/15	110/61/2/1	103/64/7	30/59/77/8	4.00±3.09	NA	NA	NA	36/138	≥2/<2 28/136	NA	NA
Kucukay 2015[8]	DEB-TACE	53	63.8±10.9	57/13	NA	NA	NA	29/19/5/0	NA	NA	NA	NA	NA	1.1±0.3	NA	NA
	cTACE	73	64.8±9.0	62/11	NA	NA	NA	29/40/4/0	NA	NA	NA	NA	NA	1.0±0.2	NA	NA
Lammer 2010 [38]	DEB-TACE	93	67.3±9.1	79/14	16/22/43/21	74/19/0/0	77/16/0	24/69/0/0	NA	8.89±5.21	-/41	NA	0/93	>3/≤3 0/93	>250/≤250 0/93	NA
	cTACE	108	67.4±8.8	95/13	18/18/57/25	80/28/0/0	89/19/0	29/79/0/0	NA	8.92±5.93	-/45	NA	0/108	>3/≤3 0/108	>250/≤250 0/108	NA
Lee 2017[32]	DEB-TACE	106	64 (38–90)	80/26	80/11/-/15	NA	85/21/0	20/77/9/0	NA	3.4 (1.3–12.1)	75/31	NA	4/102	0.7 (0.3–2.8)	31 (8–101)	21 (1–22 392)
	cTACE	144	61 (30–89)	124/20	119/14/-/11	NA	95/49/0	49/73/22/0	NA	2.5 (1.0–14.0)	86/58	NA	8/136	0.7 (0.1–2.3)	31 (11–407)	24 (1–83 000)
Megias 2015[33]	DEB-TACE	30	64.8±9.56	24/6	4/19/10/4	NA	14/16/0	NA	1.4 (1–5)	NA	-/5	NA	1/29	NA	NA	NA
	cTACE	30	61.9±10.59	21/9	5/20/10/2	NA	19/11/0	NA	1.23 (1–4)	NA	-/10	NA	4/26	NA	NA	NA
Rahman 2016[9]	DEB-TACE	45	63±13	36/9	14/5/-/14	-/-/-/0	19/26/0	9/36/0/0	2.13±1.01	7.38±4.81	45/-	45/0	NA	NA	NA	NA
	cTACE	34	61±10	26/8	13/4/-/4	-/-/-/0	11/13/0	11/23/0/0	1.44±0.82	8.95±5.87	34/-	34/0	NA	NA	NA	NA
Recchia 2012[10]	DEB-TACE	35	72 (53–80)	25/10	NA	NA	NA	NA	NA	4.12(1–9)	NA	35/0	NA	≥3/<3 0/35	≥270/<270 0/35	NA
	cTACE	70	70 (47–80)	50/20	NA	NA	NA	NA	NA	53(2–9)	NA	70/0	NA	≥3/<3 0/70	≥270/<270 0/70	NA
Song 2012[7]	DEB-TACE	60	61.7±9.8	42/18	44/8/4/4	NA	56/4/0	27/33/0/0	NA	4.2±2.8	NA	NA	NA	NA	NA	<200/≥200 48/12
	cTACE	69	59.4±11.2	48/21	46/8/12/3	NA	62/6/0	28/41/0/0	NA	5.0±3.1	NA	NA	NA	NA	NA	48/21
Hannah 2011[42]	DEB-TACE	16	67.3±9.8	14/2	4/4/-/8	9/7/0/0	14/2/0	2/9/5/0	NA	NA	NA	NA	NA	1.17±0.58	59.9±29.6	1803.4±5409.2
	cTACE	14	56.6±13.4	11/3	4/0/-/10	10/2/2/0	14/0/0	1/10/3/0	NA	NA	NA	NA	NA	1.13±0.58	52.7±29.2	8069.2±23342.3
Philipp 2011[36]	DEB-TACE	22	70.32±7.06	18/4	-/-/2/14	NA	22/0/0	1/17/3/0	2.09±1.15	7.44±3.37	NA	NA	NA	NA	NA	NA
	cTACE	22	67.72±9.02	19/3	-/-/7/12	NA	22/0/0	4/15/2/0	1.95±1.62	6.98±3.81	NA	NA	NA	NA	NA	NA
Golfieri 2014[11]	DEB-TACE	89	68.9±8.0	66/23	-/-/16/0	64/25/0/0	75/14/0	41/26/22/0	2 (1–5)^#^	3.1±1.6	-/17	NA	NA	NA	NA	NA
	cTACE	88	68.3±8.0	69/19	-/-/20/0	67/21/0/0	77/11/0	41/23/24/0	2 (1–4)^#^	3.4±1.9	-/20	NA	NA	NA	NA	NA
Sacco 2011[40]	DEB-TACE	33	71.3±7.2	23/10	4/22/-/7	NA	29/4/0	22/11/0/0	1.1	44.7±26.8	25/-	NA	NA	NA	74.1±62.2	662±1679
	cTACE	34	68.7±8.1	22/12	4/25/-/5	NA	25/9/0	22/12/0/0	1.4	38.5±18.9	28/-	NA	NA	1.25±1	54.0±38.8	67.5±202
Thomas 2010[44]	DEB-TACE	102	67.0±9.2	88/14	14/20/41/27	74/19/0/0	77/16/0	26/76/0/0	NA	NA	52/41	NA	NA	NA	NA	25.62±890.28
	cTACE	110	67.3±8.8	97/13	13/12/52/33	80/28/0/0	89/19/0	29/81/0/0	NA	NA	63/45	NA	NA	NA	NA	27.54±2400.19
Nicolini 2013[35]	DEB-TACE	22	57.2±6.5	19/3	8/10/-/4	NA	NA	14/8/0/0	NA	1.8 (0.7–4.5)	NA	NA	NA	NA	NA	>70/≤70 3/19
	cTACE	16	55.6±6.5	15/1	3/12/-/1	NA	NA	7/9/0/0	NA	2.2 (1–10)	NA	NA	NA	NA	NA	>70/≤70 5/11

* Mean±SD, # Median(range), NA Not Available, cTACE Conventional transarterial chemoembolization, DEB-TACE Drug-eluting beads, TARE Transarterial radioembolization, PVTT Portal vein tumor thrombus

### Overall survival

**Fig 2:**
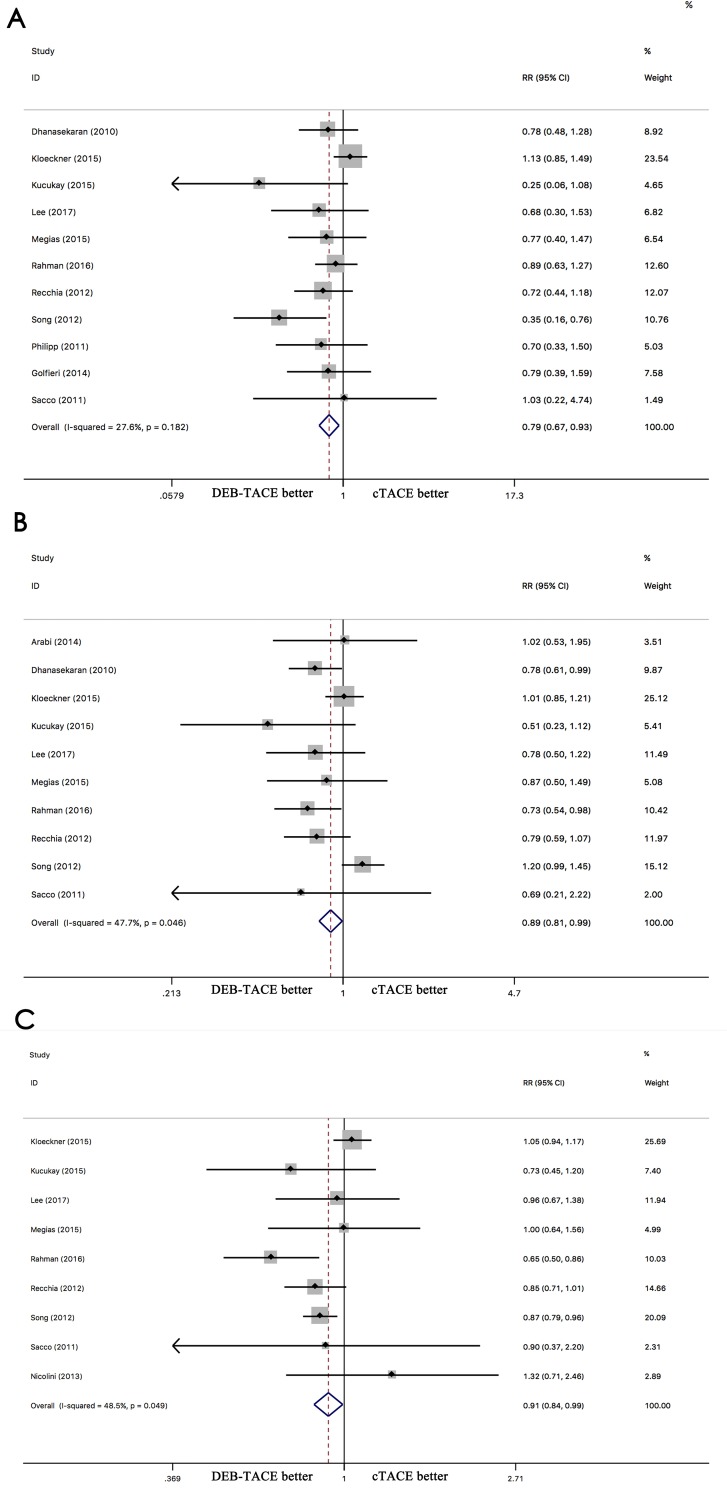
Comparison of overall survival between DEB-TACE and cTACE for hepatocellular carcinoma at 1-year(A), 2-year(B) and 3-year(C).

**Fig 3:**
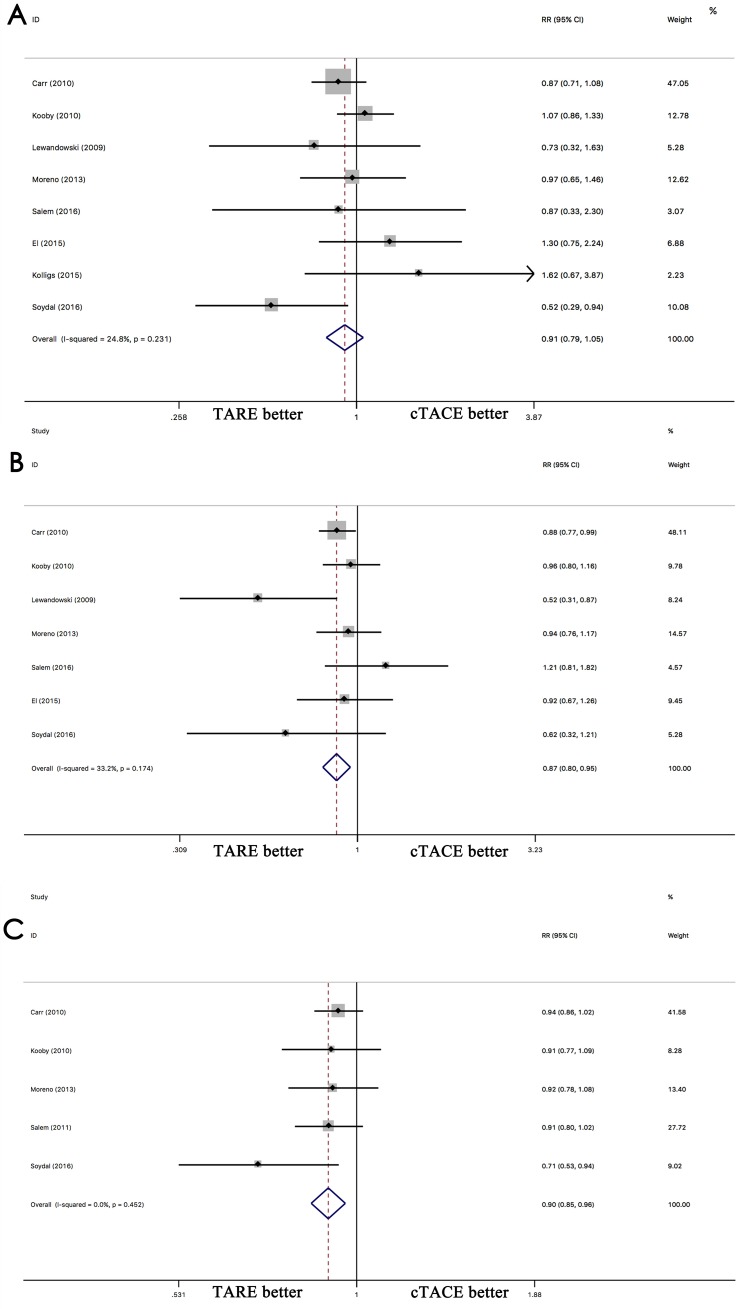
Comparison of overall survival between TARE and cTACE for hepatocellular carcinoma at 1-year(A), 2-year(B) and 3-year(C).

The comparison of DEB-TACE with cTACE indicated that DEB-TACE has a better 1-year (RR 0.79; 95% CI 0.67–0.93, p = 0.006), 2-year (RR 0.89; 95% CI 0.81–0.99, p = 0.046), and 3-year (RR 0.89; 95% CI 0.81–0.99, p = 0.035) OS than cTACE (Fig 2A–2C). The comparison of TARE with cTACE indicated that the 1-year OS is similar between TARE and cTACE (RR 0.91; 95% CI 0.79–1.05, p = 0.215), but a better OS than cTACE at 2-year (RR 0.87; 95% CI 0.80–0.95, p = 0.003) and 3-year (RR 0.90; 95% CI 0.85–0.96, p = 0.001) (Fig 3A–3C). No significant differences between DEB-TACE and TARE were found in terms of 1-year OS (RR 0.83, 95% CI 0.68–1.02, p = 0.081) (S1A Fig). DEB-TACE exhibited better 2-year OS than TARE (RR 0.40; 95% CI 0.19–0.84, p = 0.016) (S1B Fig).

**Fig 4:**
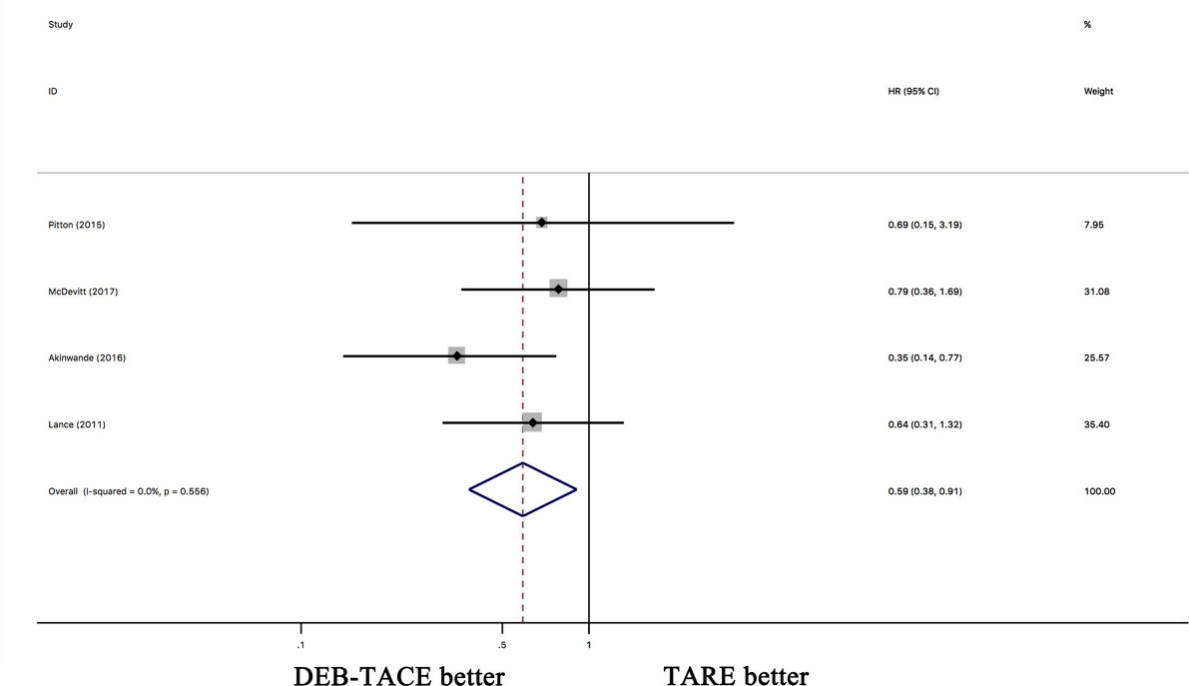
The pooled HRs for OS between DEB-TACE and TARE for hepatocellular carcinoma.

However, when the HRs were pooled, DEB-TACE showed a similar OS as cTACE (HR 0.98; 95% CI 0.81–1.18, p = 0.144) (S2A Fig). The pooled HRs indicated that TARE was superior to cTACE regarding OS (HR 0.84; 95% CI 0.70–1.00, p = 0.049) (S2B Fig). The pooled HRs indicated that DEB-TACE had a better OS than TARE (HR 0.59; 95% CI 0.38–0.91, p = 0.016) (Fig 4).

### Tumor response

An objective response was defined as a complete response plus a partial response, and the disease control rate (DCR) was defined as an objective response plus stable disease. For objective response, no significant difference was observed between DEB-TACE and cTACE (OR 0.99; 95% CI 0.73–1.34, p = 0.926). In contrast, TARE was superior to cTACE (OR 0.77; 95% CI 0.57–1.03, p = 0.082). No difference in the DCR was found between DEB-TACE and cTACE (OR 1.39; 95% CI 0.96–2.01, p = 0.079). TARE had a better DCR than cTACE (OR 1.89; 95% CI 1.07–3.35, p = 0.029). However, no significant difference regarding the DCR was found between DEB-TACE and cTACE (OR 1.39; 95% CI 0.96–2.01, p = 0.079).

### Progression free survival and time to progress

In terms of progression free survival (PFS), there were no significant differences between DEB-TACE and cTACE at 1 year (RR 0.75; 95% CI 0.54–1.03, p = 0.076), 2 years (RR 0.83; 95% CI 0.67–1.03, p = 0.092), or 3 years (RR 0.99; 95% CI 0.85–1.15, p = 0.885). There were also no significant differences between DEB-TACE and TARE at 1 year (RR 1.00; 95% CI 0.80–1.25, p = 1.000). The TTP was not significantly different between DEB-TACE and cTACE at 1 year (RR 1.1; 95% CI 0.89–1.36, p = 0.385).

### Adverse events

The main adverse events, including nausea/vomiting, pain, fatigue, infection/fever, liver failure, and gastrointestinal bleeding, are presented in S3 Table. In the comparison of DEB-TACE and cTACE, significant differences were observed for fatigue (OR 9.00 95% CI 3.99–20.31, p = 0.000) and infection/fever (OR 0.45 95% CI 0.23–0.91, p = 0.027). The other complications are presented in S3 Table. The graded adverse events are presented in S4 Table.

### Meta-regression analysis

**Fig 5:**
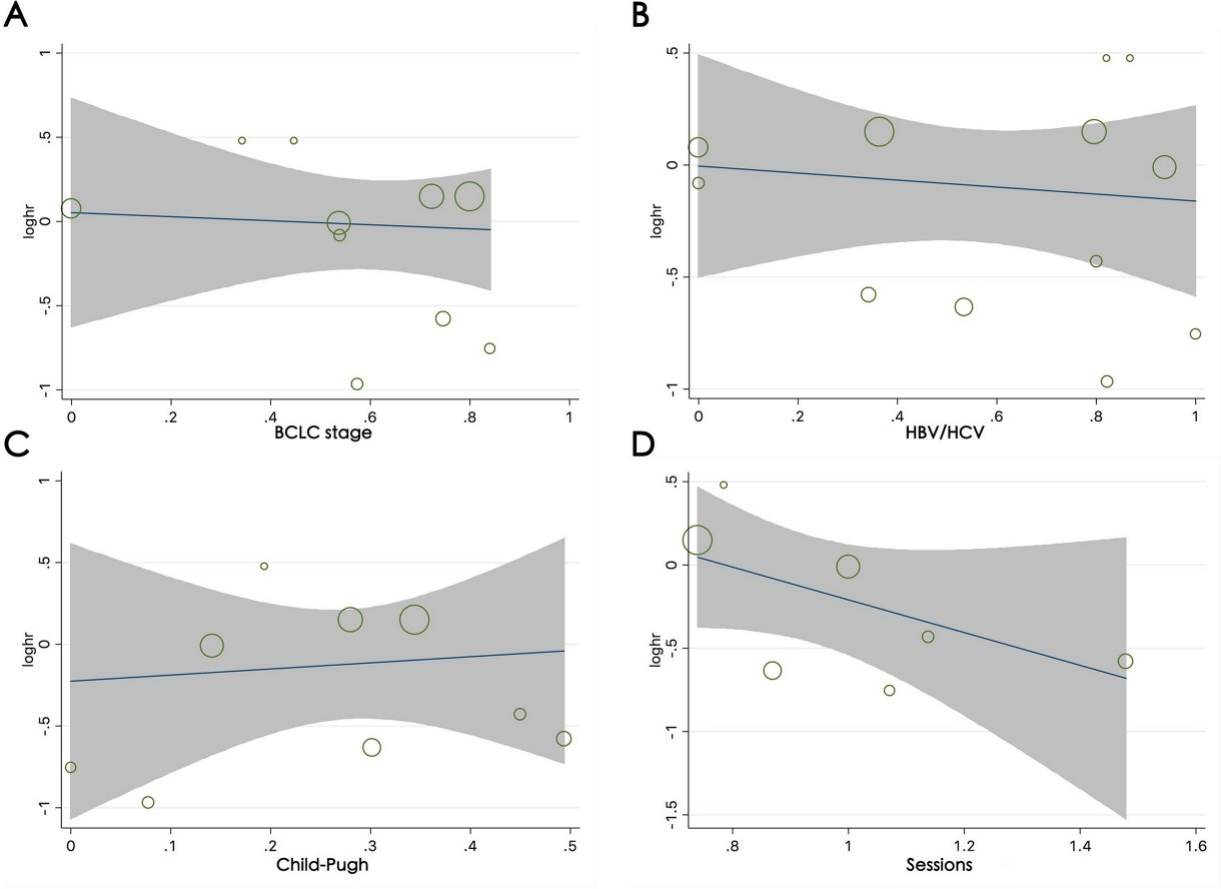
Meta-regression analysis for OS between DEB-TACE and cTACE. Bubble plot with a fitted meta-regression line of the log HR for (A) the baseline proportion of BCLC-B/C, (B) the baseline proportion of HBV/HCV infection, (C) the baseline proportion of Child-Pugh classification A, (D) and the number of treatment sessions. The size of the circles is proportional to the weight of each study in the fitted random-effects meta-regression.

The meta-regression analysis for DEB-TACE vs. cTACE and TARE vs. cTACE showed a trend for study design, baseline proportion of HBV/HCV infection, BCLC stage, Child-Pugh classification and number of treatment sessions, but the results were not statistically significant (for all p > 0.05; Fig 5A–5D).The contribution of the different study characteristics to the level of heterogeneity in the terms of overall effect estimates was calculated (S5 Table). No significant factors contributed to the observed heterogeneity or to the proportion of heterogeneity (for all covariates, p > 0.05). The meta-regression analysis categorized by BCLC stage and number of treatment sessions for TARE vs. cTACE and by all factors for DEB-TACE vs. cTACE were not performed due to a lack of data in the included studies.

### Publication bias

No publication bias was found via Egger's (p = 0.11; 95% CI -3.30–0.39) and Begg's tests (Z = 0.18; p = 0.86) for DEB-TACE vs. cTACE. The results of both Egger's (p = 0.288; 95% CI -11.13–8.03) and Begg's tests (Z = 0.00; p = 1.00) in the comparison of DEB-TACE with TARE and Egger's (p = 0.57; 95% CI -3.06–1.90) and Begg’s (Z = 0.60; p = 0.55) tests in the comparison of DEB-TACE with TARE showed no publication bias (S3 Fig).

## Discussion

We performed this study to compare the efficacy DEB-TACE with cTACE, TARE-TACE with cTACE and DEB-TACE with TARE in patients with HCC. Our results indicated that DEB-TACE has a better 1-, 2-, and 3-year OS than cTACE, TARE has a similar 1-year and better 2-year and 3-year OS as cTACE, and DEB-TACE and TARE have a similar 1-year OS. However, compared with TARE, DEB-TACE showed a longer OS when the follow-up time was prolonged to 2 years. Additionally, pooling the HRs in the comparison of DEB-TACE with TARE indicated that DEB-TACE had a better OS than TARE, whereas no significant differences were observed in the comparison of DEB-TACE vs. cTACE or in the comparison of TARE vs. cTACE regarding an objective response. Compared with cTACE, TARE showed a higher DCR. No significant differences were observed in the comparison of DEB-TACE vs. cTACE or in the comparison of TARE vs. cTACE in terms of PFS. However, compared with cTACE, DEB-TACE showed significantly lower rates of fatigue and infection/fever. As the complications based on different criteria for adverse events in the including studies. Hence, we did not pool the complications.

Previously, a meta-analysis comparing DEB-TACE with cTACE performed by Facciorusso et al[45] showed in the 1-year OS (OR 0.76, 95% CI 0.48–1.21, p = 0.25), 2-year OS (OR 0.68, 95% CI 0.42–1.12, p = 0.13), and 3-year OS (OR 0.57, 95% CI 0.32–1.01, p = 0.06)(S6 Table). Chen et al[46] reported that patients in DEB-TACE group received significantly higher 1-, 2-, and 3-year OS rates with pooled RRs of 1.12 (95% CI 1.03–1.23, p = 0.007), 1.26 (95% CI 1.03–1.54, p = 0.02), and 1.69 (95% CI 1.00–2.84, p = 0.04). These results are consistent with our results. Facciorusso et al[45] also revealed no statistically significant differences regarding the occurrence of severe adverse events (OR 0.85, 95% CI 0.60–1.20, p = 0.36) and an objective response (OR 1.21, 95% CI 0.69–2.12, p = 0.51) between DEB-TACE and cTACE. Chen et al[46] additionally found no statistically significant differences in the occurrence of an objective response (RR 1.09; 95% CI = 0.94–1.25, p = 0.25), DCR (RR 1.09; 95%CI 0.94–1.25, p = 0.25), postembolization syndrome (RR 0.87; 95% CI 0.71–1.07, p = 0.19) or liver dysfunction (RR 0.91; 95%CI 0.25–3.23, p = 0.88) in the DEB-TACE group compared with the cTACE group. In our study, DEB-TACE had no effect on the rate of an objective response or DCR compared with cTACE for HCC. These results are similar with another study performed by Zou et al[3]. In our study, a significant improvement with regards to the occurrence of fatigue and infection/fever was found for DEB-TACE compared with cTACE. Zou et al[3] reported fewer common adverse events using DEB-TACE than with cTACE (OR 0.59, 95% CI 0.41–0.84).

Two meta-analyses have been performed comparing TARE with cTACE. Lobo et al[47] reported no differences between TARE and cTACE for 1- and 3-year OS. At 2 years, TARE statistically significantly increased the OS compared with cTACE (RR 1.36; 95% CI 1.05–1.76; p = 0.02) (S6 Table). Our study showed no difference between TARE and cTACE in terms of the 1-year OS, and the 2- and 3-year OS was significantly better for TARE than for TACE, which is consistent with another study performed by Zhang et al[48]. In our study, the DCR was higher for TARE than for cTACE. This is similar to the results of Zhang et al[48]. Lobo et al[47] found more patients with fatigue with TARE than with TACE (RR 1.68; 95% CI 1.08–2.62; p = 0.01), and Zhang et al[48] found that TARE led to a higher incidence of lower abdominal pain (RR 0.30; 95% CI 0.11–0.83; p = 0.02) than TACE. No significant differences in the incidence of nausea and vomiting and fever have been observed, which is consistent with our study findings. To easily understand the research status of this subject, we summarized the meta studies in S6 Table [3, 45–52].

Previously, no meta-analysis directly compared the efficacy and safety of DEB-TACE or TARE with TACE in HCC patients. Ludwig et al[50] performed a meta-analysis to indirectly compare DEB-TACE with TARE(S6 Table). The 1-year OS was significantly increased with DEB-TACE (OR 0.57; 95% CI 0.36–0.92). No statistically significant22 benefit was observed for DEB-TACE over TARE in terms of the 2-year (OR 0.65; 95% CI 0.29–1.44) or 3-year OS (OR 0.71; 95% CI 0.21–2.55). These results are opposite to our results. Ludwig et al[50] also admitted the limitations of insufficient studies. The statistical analysis of the overall response showed no significant difference, which is consist with our results. In terms of the survival rate, Akinwande et al [19] showed a significantly increased OS in the DEB-TACE group, with an OS of 13 months with DEB-TACE vs. 4 months with TARE. This result is consistent with their previous results [53]. The other two studies demonstrated no significant difference between DEB-TACE and TARE regarding OS[21, 39]. While in these studies, there are more TACE patients with BCLC stage A (42% TACE vs. 20% TARE). Moreover, Comparing with DEB-TACE patients, more TARE patients have three or more tumor lesions (42% TACE vs. 69%TARE)(S7 Table). Patients with portal vein tumor thrombus benefited from a longer overall survival with DEB-TACE (6 months) than with TARE (3 months, p = 0.13)[19]. Pitton et al[39] reveled that there was no difference between DEB-TACE and TARE regarding PFS (7.2 vs. 6 months). This is consistent with another study (DEB-TACE vs. TARE: 6 vs. 5 months; p = 0.42)[19]. Pitton et al[39] also showed no significant difference between DEB-TACE and TARE with respect to time to progression (371 days after TARE versus 336 days after DEB-TACE). Akinwande et al [19] showed that DEB-TACE provides superior DCR compared to TARE. This is mainly due to higher rates of stable disease in Akinwande's study[19]. In terms of complications, Lance et al[20] showed that the postembolization syndrome rate was similar between DEB-TACE and TARE. Also, no significant differences in major or minor complication rates associated with causes other than postembolization syndrome were observed(p = 0.58). Lance et al[20] demonstrated that the degree of postembolization syndrome severity was significantly worse in the DEB-TACE patients (p = 0.02). Pitton et al[39] also revealed that the reduced number of TARE treatment sessions and hospital days might be a significant difference that reflects an advantage in terms of quality of life. This finding can be explained by less vessel damage for TARE[39]. However, both McDevitt et al[21] and Akinwande et al[53] showed no differences between the groups in terms of the incidence of high-grade side effects. McDevitt et al[21] showed that immediate low-grade clinical toxicities after both procedures, although patients treated with TARE were significantly less likely to report abdominal pain (p = 0.004) or fever or chills (p = 0.01).

### Limitations of this study

We acknowledge several limitations of this study. First, a small number of relevant studies were included. Second, both retrospective studies and randomized controlled trials were included, which might cause a potential bias. Third, as lacking of original data, part of the data were extracted from the survival curves. Although we tried our best to extract data using the software Engauge Digitizer, some errors are inevitable. While, the Cochrane Collaborative Group allows that some data are not perfectly accurate when the original data are lacking[54]. Fourth, the included studies differed slightly in their study designs and definitions for study outcomes. Some credible techniques were carefully performed to decrease the potential bias, such as the use of clear criteria, an extensive search of the literature, strict guidelines regarding duplicate data extraction, and contacting the corresponding authors by email. Although all of the above-described limitations, this study still provides the most comprehensive comparison of DEB-TACE, TARE, and cTACE.

## Conclusion

The current meta-analysis suggests that both DEB-TACE and TARE are superior to cTACE in terms of OS and complications. DEB-TACE has significantly better OS rates for patients with HCC than TARE. Further multicenter, well-designed randomized trials are needed, especially to compare DEB-TACE with TARE.

## Supporting information

S1 Fig (A) Comparison of the overall survival between DEB-TACE and TARE for hepatocellular carcinoma at 1 year. (B) Comparison of the OS between DEB-TACE and TARE for hepatocellular carcinoma at 1 year.
(TIF)

S2 Fig The pooled HR according to OS between DEB-TACE vs. cTACE(A) and TARE vs. cTACE(B) for hepatocellular carcinoma.
(TIF)

S3 Fig The funnel plots for publication bias of cTACE vs. TARE (90Y) including 1-year overall survival rate group. (A) The bias of DEB-TACE vs. cTACE, (B). The bias of TARE vs. cTACE, (C) The bias of DEB-TACE vs. TARE.
(TIF)

S1 Table Basic characteristics of each study. (DOCX)

S2 Table The endpoints of transarterial therapies for hepatocellular carcinoma. (DOCX)

S3 Table The comparison of main adverse events in the transarterial therapies for hepatocellular carcinoma. (DOCX)

S4 Table Summary of graded adverse events of transarterial therapies for hepatocellular carcinoma. (DOCX)

S5 Table Meta-regression analysis for overall survival. (DOCX)

S6 Table Key meta studies for TARE, DEB-TACE, and cTACE in the treatment of unresectable liver cancer. (DOCX)

S7 Table Key limitations for comparison of radioembolisation and DEB-TACE in the treatment of unresectable liver cancer. (DOCX)

S1 File PRISMA 2009 checklist. (DOC)

S2 File Search strategy in PubMed. (DOCX)
