# Correction: Transarterial strategies for the treatment of unresectable hepatocellular carcinoma: A systematic review

**DOI:** 10.1371/journal.pone.0230369

**Published:** 2020-03-06

**Authors:** Biao Yang, Jie Liang, ZiYu Qu, FangYun Yang, ZhengYin Liao, HongFeng Gou

There are errors in the author affiliations. The correct affiliations are as follows:

Biao Yang^1,2^, Jie Liang^3^, ZiYu Qu^2^, FangYun Yang^2^, ZhengYin Liao^2^, HongFeng Gou^2^

**1** Department of Gastroenterology, West China Hospital, West China Medical School, Sichuan University, Chengdu, P.R. China, **2** Department of Abdominal Oncology, West China Hospital, West China Medical School, Sichuan University, Chengdu, P.R. China, **3** School of Public Health, Chengdu University of Traditional Chinese Medicine, Chengdu, P.R. China.
