# Supplementary figures and images for: Transarterial strategies for the treatment of unresectable hepatocellular carcinoma: A systematic review
Source: PLoS One. 2020 Feb 19;15(2):e0227475. doi: 10.1371/journal.pone.0227475 (PMC7029952; doi:10.1371/journal.pone.0227475)

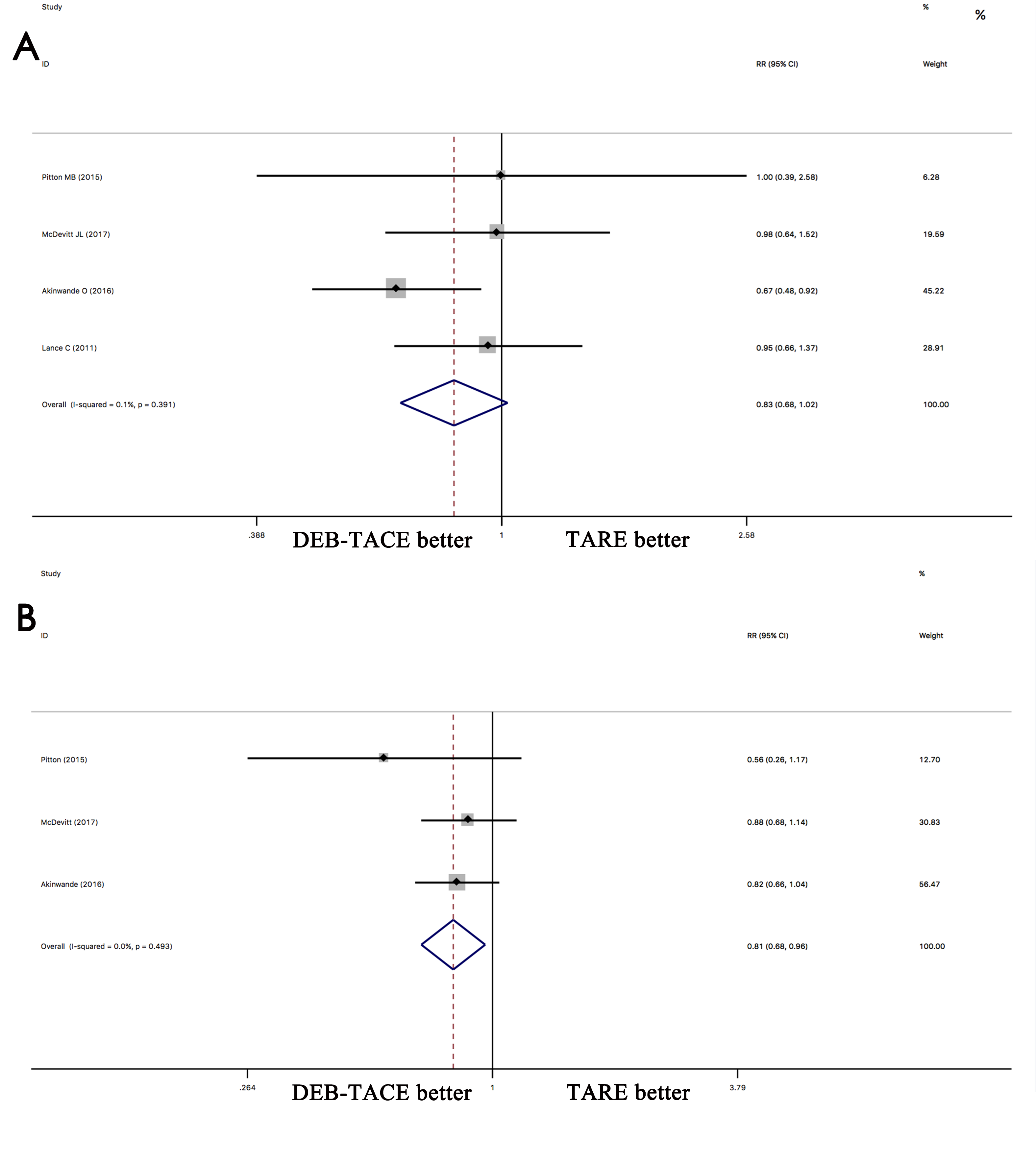

Supplement: S1 Fig — (A) Comparison of the overall survival between DEB-TACE and TARE for hepatocellular carcinoma at 1 year. (B) Comparison of the OS between DEB-TACE and TARE for hepatocellular carcinoma at 1 year. (TIF) [file pone.0227475.s001.tif]

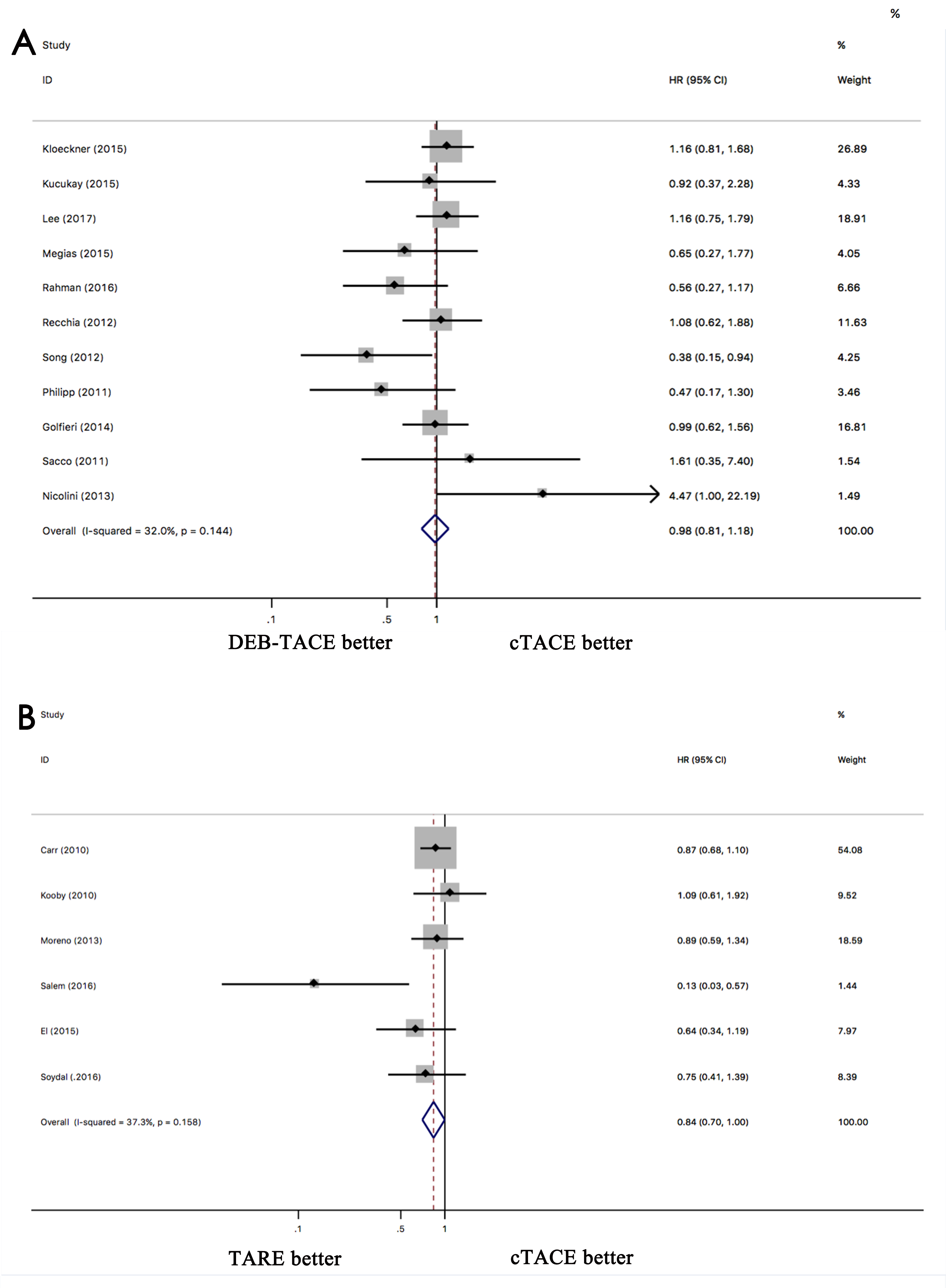

Supplement: S2 Fig — The pooled HR according to OS between DEB-TACE vs. cTACE(A) and TARE vs. cTACE(B) for hepatocellular carcinoma. (TIF) [file pone.0227475.s002.tif]

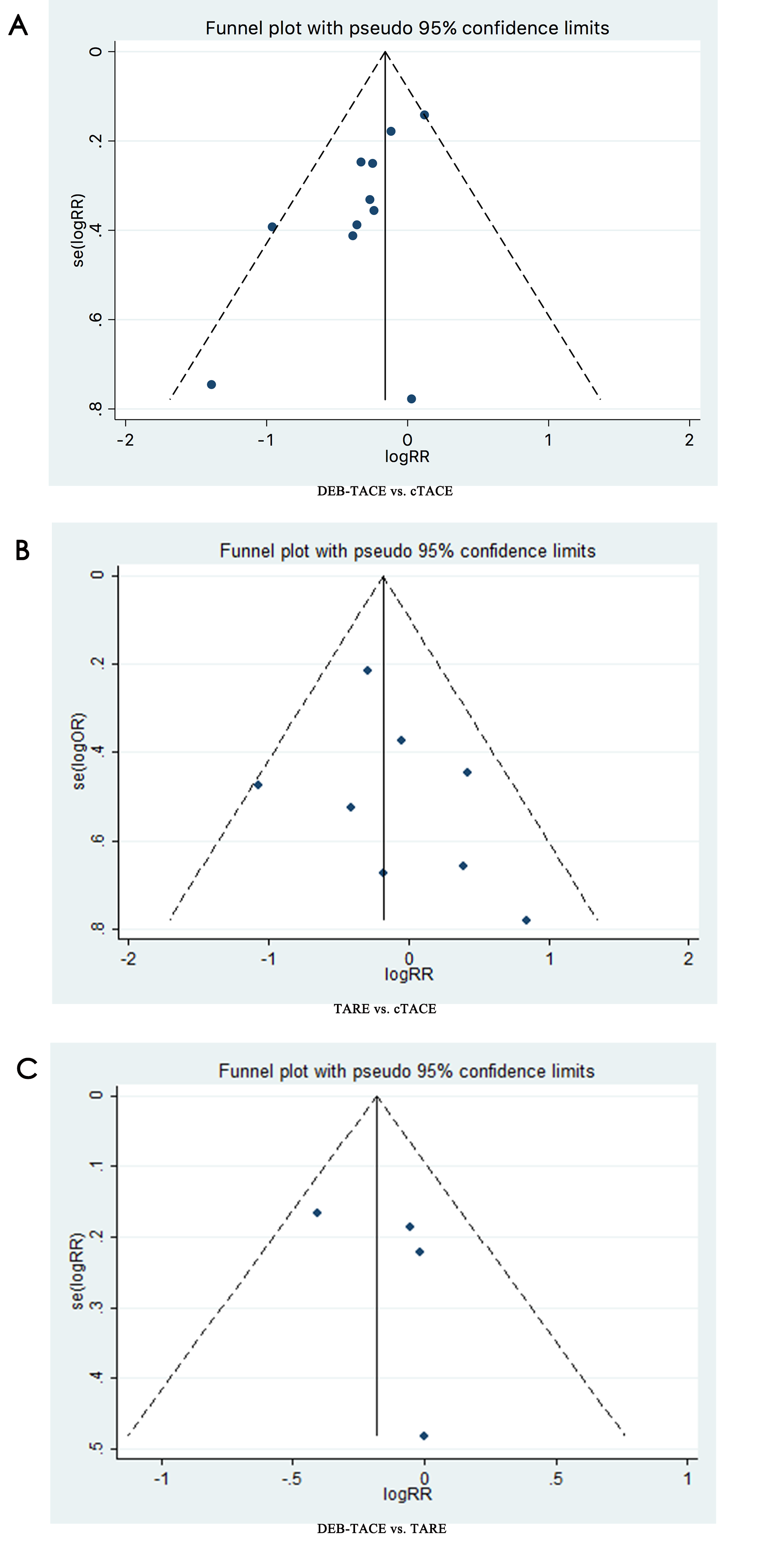

Supplement: S3 Fig — The funnel plots for publication bias of cTACE vs. TARE (90Y) including 1-year overall survival rate group. (A) The bias of DEB-TACE vs. cTACE, (B). The bias of TARE vs. cTACE, (C) The bias of DEB-TACE vs. TARE. (TIF) [file pone.0227475.s003.tif]
