# Supplementary material for: Transarterial strategies for the treatment of unresectable hepatocellular carcinoma: A systematic review
Source: PLoS One. 2020 Feb 19;15(2):e0227475. doi: 10.1371/journal.pone.0227475 (PMC7029952; doi:10.1371/journal.pone.0227475)
Supplement: S1 Table — (DOCX) [file pone.0227475.s004.docx]

S1 Table: Basic characteristics of each study

| First Author | Treatment | Number(n) | Duration | Region(s) | Center(s) | Study design | Newcastle-Ottawa score |
| --- | --- | --- | --- | --- | --- | --- | --- |
| Pitton 2015 | DEB-TACE | 12 | Apr.2010 - Jul.2012 | Germany | Single | RCT | 4 + |
|  | TARE | 12 |  |  |  |  |  |
| McDevitt 2017 | DEB-TACE | 26 | Mar. 2007 - Aug. 2012 | U.S.A | Single | Retrospective | 7 |
|  | TARE | 24 |  |  |  |  |  |
| Akinwande 2016 | DEB-TACE | 48 | 2007 - 2013 | U.S.A | Multiple | Prospective | 7 |
|  | TARE | 48 |  |  |  |  |  |
| Lance 2011 | DEB-TACE | 35 | Aug. 2007 - Apr. 2010 | U.S.A | Single | Retrospective | 6 |
|  | TARE | 38 |  |  |  |  |  |
| Carr 2010 | Y90 | 99 | 1992 - 2005 | U.S.A | Single | Retrospective | 7 |
|  | cTACE | 691 |  |  |  |  |  |
| Kooby 2010 | Y90 | 27 | Jan. 1996 - Dec. 2006 | U.S.A | Single | Retrospective | 7 |
|  | cTACE | 44 |  |  |  |  |  |
| Lewandowski 2009 | Y90 | 43 | Jan. 2000 - Dec. 2008 | U.S.A | Single | Retrospective | 7 |
|  | cTACE | 43 |  |  |  |  |  |
| Moreno 2013 | Y90 | 61 | Apr. 1998 - Aug. 2007 | U.S.A | Single | Retrospective | 7 |
|  | cTACE | 55 |  |  |  |  |  |
| Salem 2016 | Y90 | 24 | Oct. 2009 - Oct. 2015 | U.S.A | Single | RCT | 5+ |
|  | cTACE | 21 |  |  |  |  |  |
| El 2015 | Y90 | 44 | Nov. 2009 - Oct. 2011 | Germany,Egypt | Multiple | Prospective | 7 |
|  | cTACE | 42 |  |  |  |  |  |
| Kolligs 2015 | Y90 | 13 | Jul. 2007 - Jun. 2011 | Germany,Spanish | Multiple | RCT | 4+ |
|  | cTACE | 15 |  |  |  |  |  |
| Soydal 2016 | Y90 | 40 | Jun. 2008 - Nov. 2014 | Turkey | Single | Retrospective |  |
|  | cTACE | 40 |  |  |  |  |  |
| Arabi 2014 | DEB-TACE | 35 | 2006 - 2014 | Saudi Arabia | Single | Retrospective | 7 |
|  | cTACE | 19 |  |  |  |  |  |
| Dhanasekaran 2010 | DEB-TACE | 45 | Jan. 1998 - Jul. 2008 | U.S.A | Single | Retrospective | 8 |
|  | cTACE | 26 |  |  |  |  |  |
| Kloeckner 2015 | DEB-TACE | 76 | Nov. 2002 - Dec. 2013 | Germany | Single | Retrospective | 6 |
|  | cTACE | 174 |  |  |  |  |  |
| Kucukay 2015 | DEB-TACE | 53 | Jan. 2007 - Oct. 2014 | Turkey | Single | Retrospective | 7 |
|  | cTACE | 73 |  |  |  |  |  |
| Lammer 2010 | DEB-TACE | 93 | Nov. 2005 - Jun. 2007 | France | Multiple | RCT | 4+ |
|  | cTACE | 108 |  |  |  |  |  |
| Lee 2017 | DEB-TACE | 106 | Jan. 2010 - Apr.2014 | Korea | Single | Retrospective | 7 |
|  | cTACE | 144 |  |  |  |  |  |
| Megias 2015 | DEB-TACE | 30 | 2008 - 2009 | Spain | Single | Retrospective | 7 |
|  | cTACE | 30 |  |  |  |  |  |
| Rahman 2016 | DEB-TACE | 45 | Jan. 2009 - Dec. 2014 | Malaysia | Single | Retrospective | 7 |
|  | cTACE | 34 |  |  |  |  |  |
| Recchia 2012 | DEB-TACE | 35 | Jan. 2008 - Dec. 2010 | Italy | Single | Prospective | 8 |
|  | cTACE | 70 |  |  |  |  |  |
| Song 2012 | DEB-TACE | 60 | Aug. 2008 - Feb. 2011 | Korea | Single | Retrospective | 9 |
|  | cTACE | 69 |  |  |  |  |  |
| Hannah 2011 | DEB-TACE | 16 | Sept. 2006 - Feb. 2009 | Belgium | Single | RCT | 4+ |
|  | cTACE | 14 |  |  |  |  |  |
| Philipp 2011 | DEB-TACE | 22 | 2003 - 2008 | Germany | Single | Retrospective | 8 |
|  | cTACE | 22 |  |  |  |  |  |
| Golfieri 2014 | DEB-TACE | 89 | Mar. 2008 - Dec. 2010 | Italy | Multiple | RCT | 5+ |
|  | cTACE | 88 |  |  |  |  |  |
| Sacco 2011 | DEB-TACE | 33 | Jan. 2006 - Mar. 2009 | Italy | Single | RCT | 4+ |
|  | cTACE | 34 |  |  |  |  |  |
| Thomas 2010 | DEB-TACE | 102 | Nov. 2005 - Jun. 2007 | U.S.A, Italy | Multiple | RCT | 4+ |
|  | cTACE | 110 |  |  |  |  |  |
| Nicolini 2013 | DEB-TACE | 22 | Aug. 2005 - Mar. 2011 | Italy | Single | Retrospective | 6 |
|  | cTACE | 16 |  |  |  |  |  |
| Zhou 2019 | DEB-TACE | 74 |  | China | Single | Retrospective | 6 |
|  | cTACE | 80 |  |  |  |  |  |
| Zhang 2018 | DEB-TACE |  | Nov. 2015 - Nov. 2016 | China | Single | Prospective | 7 |
|  | cTACE |  |  |  |  |  |  |

RCT Randomized control trails, + Jade Score, cTACE Conventional transarterial chemoembolization, DEB-TACE Drug-eluting beads, TARE Transarterial radioembolization
