# Supplementary material for: Transarterial strategies for the treatment of unresectable hepatocellular carcinoma: A systematic review
Source: PLoS One. 2020 Feb 19;15(2):e0227475. doi: 10.1371/journal.pone.0227475 (PMC7029952; doi:10.1371/journal.pone.0227475)
Supplement: S2 Table — (DOCX) [file pone.0227475.s005.docx]

S2 Table: The endpoints of transarterial therapies for hepatocellular carcinoma

| First author | Treatment | Follow-up^*^ (months) | Mean survival^*^ (months) | Tumor response(%) | | | | Overall survival rate (%) | | | Multivariate analysis (HR 95%CI) |
| --- | --- | --- | --- | --- | --- | --- | --- | --- | --- | --- | --- |
|  |  |  |  | CR | PR | SD | PD | 6-m | 1-y | 2-y |  |
| Pitton 2015 | DEB-TACE | 12.47±10.13 | 26.3 (5.9-31.7) | NA | NA | NA | NA | 75 | 58.3 | 58.3 | 0.69 (0.15-3.19) |
|  | TARE | 14.5±10.67 | 19.73 (6.4/–) | NA | NA | NA | NA | 75 | 58.3 | 25 |  |
| McDevitt 2017 | DEB-TACE | 9.8 (1.2–49)^#^ | 9.8 (1.2–49) | NA | NA | NA | NA | 73.1 | 40 | 24 | 0.79 (0.36-1.69) |
|  | TARE | 8.1 (1.1–27)^#^ | 8.1 (1.1–27) | NA | NA | NA | NA | 62.5 | 37 | 13 |  |
| Akinwande 2016 | DEB-TACE | NA | 13 | 13 | 34 | 26 | 27 | 64.6 | 50 | 31.3 | 0.35 (0.14 -0.77) |
|  | TARE | NA | 4 | 11 | 24 | 13 | 52 | 35.4 | 25 | 16.7 |  |
| Lance 2011 | DEB-TACE | 16.4^#^ | 10.3 | NA | NA | NA | NA | 62.9 | 40 | NA | 0.64 (0.31-1.32) |
|  | TARE | 12.1 | 8.0 | NA | NA | NA | NA | 65.8 | 36.8 | NA |  |
| Carr 2010 | TARE | NA | 11.5 (8-10)^§^ | 3.03 | 38.38 | 35.35 | 23.23 | 71.7 | 50.5 | 26.3 | 0.87(0.68-1.10) |
|  | cTACE | NA | 8.5(8-16)^§^ | 5.35 | 54.99 | 28.8 | 10.85 | 61.5 | 43.4 | 15.8 |  |
| Kooby 2010 | TARE | 6 | 11.2±2.8 | 0 | 11 | 41 | 33 | 55.6 | 16 | 14.8 | 1.09(0.61-1.92) |
|  | cTACE | 6 | 9.8±1.9 | 2 | 4 | 36 | 36 | 50 | 20 | 11.4 |  |
| Lewandowsk 2009 | TARE | 34.1 | 41.6 (29.6,–)^§^ | 0 | 26 | 16 | 1 | NA | 81 | 69 | NA |
|  | cTACE | 51.9 | 19.2(14.7-26.5)^§^ | 0 | 37 | 49 | 5 | NA | 75 | 42 |  |
| Moreno 2013 | TARE | NA | NA | 12 | 39 | 39 | 9 | 82 | 55.7 | 27.9 | 0.89(0.59-1.34) |
|  | cTACE | NA | NA | 4 | 47 | 34 | 15 | 81.8 | 54.5 | 23.6 |  |
| Salem 2016 | TARE | 21.0 (2.3–59.6) | 18.6 ( 7.4-32.5) | 0 | 31.82 | 63.64 | 4.55 | 79.2 | 75 | 25 | 0.13 (0.03–0.57) |
|  | cTACE | 15.7 (1.4–62.1) | 17.7 (8.3-NA) | 0 | 33.33 | 66.67 | 0 | 85.7 | 71.4 | 38.1 |  |
| El 2015 | TARE | 8.5 | 16.4 ( 7.9–25.3)^§^ | 7 | 68 | 18 | 7 | 72.7 | 56.8 | 38.6 | 0.64(0.34-1.19) |
|  | cTACE | 10 | 18 (12.1–25.5)^§^ | 5 | 45 | 21 | 29 | 81 | 66.7 | 33.3 |  |
| Kolligs 2015 | TARE | 10.7 | NA | 0 | 30.8 | 46.2 | 15.4 | 69.2 | 46.2 | NA | NA |
|  | cTACE | 10.7 | NA | 0 | 13.3 | 60 | 20 | 86.7 | 66.7 | NA |  |
| Soydal 2016 | TARE | 53±15.7 | 32 (24.16-39.83)^§^ | NA | NA | NA | NA | 80 | 72 | 74 | 0.75(0.41-1.39) |
|  | cTACE | 53±15.7 | 48 (34.95-61.04)^§^ | NA | NA | NA | NA | 77.5 | 47 | 59 |  |
| Arabi 2014 | DEB-TACE | 2.03(0.7–17.9) | NA | 11 | 24 | 17 | 47 | NA | NA | 58 | NA |
|  | cTACE | 2.87(0.1–5.07) | NA | 4 | 32 | 28 | 36 | NA | NA | 60 |  |
| Dhanasekaran 2010 | DEB-TACE | NA | 20.3 (11.7–28.9)^§^ | NA | NA | NA | NA | 71 | 58 | 31 | 0.53(0.29-0.96) |
|  | cTACE | NA | 9.47(0.13–18.7)^§^ | NA | NA | NA | NA | 50 | 48 | 12 |  |
| Kloeckner 2015 | DEB-TACE | NA | 12.3(10.3–19.6)^§^ | NA | NA | NA | NA | 78.9 | 50 | 28.9 | 1.16(0.81-1.68) |
|  | cTACE | NA | 13.6（10.7-16.3) | NA | NA | NA | NA | 79.9 | 55.7 | 29.9 |  |
| Kucukay 2015 | DEB-TACE | NA | 37.4±3.3 | NA | NA | NA | NA | NA | 95.9 | 92.3 | 0.92(0.37-2.28) |
|  | cTACE | NA | 39.0±3.9 | NA | NA | NA | NA | NA | 84.9 | 74.6 |  |
| Lammer 2010 | DEB-TACE | NA | NA | 26.8 | 24.73 | 11.83 | 32.26 | NA | NA | NA | NA |
|  | cTACE | NA | NA | 22.2 | 21.3 | 8.34 | 40.74 | NA | NA | NA |  |
| Lee 2017 | DEB-TACE | NA | 46.6 | 59.4 | 18.9 | 17.9 | 3.8 | 99.1 | 92.5 | 78.3 | 1.16 (0.75-1.79) |
|  | cTACE | NA | 44.9 | 47.9 | 38.9 | 4.9 | 8.3 | 94.4 | 88.9 | 72.2 |  |
| Megias 2015 | DEB-TACE | NA | 30.67±4.09 | NA | NA | NA | NA | 80 | 66.7 | 56.7 | 0.65(0.27-1.77) |
|  | cTACE | NA | 29.99±4.39 | NA | NA | NA | NA | 80 | 56.7 | 50 |  |
| Rahman 2016 | DEB-TACE | 11.8 | 8.3±2.0 | NA | 22 | 22 | 39 | 71.1 | 42.2 | 42.2 | 0.56(0.27-1.17) |
|  | cTACE | 11.8 | 4.9±3.2 | 10 | 19 | 19 | 52 | 47.1 | 35.3 | 20.6 |  |
| Recchia 2012 | DEB-TACE | 14.1 (6-40) | 18.4 | 20 | 43 | 26 | 11 | 82.9 | 62.9 | 40 | 1.08(0.62-1.88) |
|  | cTACE | 14.1 (6-40) | 11.4 | NA | NA | NA | NA | 78.6 | 48.6 | 24.3 |  |
| Song 2012 | DEB-TACE | 18 (5.0–37.2) | 32.2±1.9 | 55 | 26.6 | 15 | 3.4 | 93 | 88 | 16.7 | 0.38 (0.15-0.94) |
|  | cTACE | 18 (5.0–37.2) | 24.7±1.7 | 23.1 | 26.3 | 20.4 | 20.2 | 80 | 67 | 30 |  |
| Hannah 2011 | DEB-TACE | NA | NA | NA | NA | 77 | 23 | NA | NA | NA | NA |
|  | cTACE | NA | NA | NA | NA | 92 | 8 | NA | NA | NA |  |
| Philipp 2011 | DEB-TACE | NA | 21.7±2.53 | 13.6 | 9.1 | 68.2 | 9.1 | NA | 70 | NA | 0.47(0.17-1.30) |
|  | cTACE | NA | 13.8±1.43 | 0 | 22.7 | 45.5 | 31.8 | NA | 55 | NA |  |
| Golfieri 2014 | DEB-TACE | NA |  | 55.7 | 19 | 5 | 20.3 | 97.7 | 86.2 | 56.8 | 0.99 (0.62-1.56) |
|  | cTACE | NA | NA | 58 | 16.1 | 3.7 | 22.2 | 93.2 | 83.5 | 55.4 |  |
| Sacco 2011 | DEB-TACE | 26.4±11.7 | NA | 51.5 | 48.5 | NA | NA | 97 | 90.9 | 86.8 | 1.61(0.35-7.4) |
|  | cTACE | 27.2±12.2 | NA | 70.6 | 29.4 | NA | NA | 97.1 | 91.2 | 83.6 |  |
| Thomas 2010 | DEB-TACE | NA | NA | NA | NA | NA | NA | NA | NA | NA | NA |
|  | cTACE | NA | NA | NA | NA | NA | NA | NA | NA | NA |  |
| Nicolini 2013 | DEB-TACE | 34.9±19.0 | NA | 44.7 | 7.9 | NA | NA | 100 | 100 | 95.5 | 4.47 (1.00-22.19)) |
|  | cTACE | 46.8±25.6 | NA | 32 | 34 | NA | NA | 100 | 87.5 | 62.5 |  |

* Mean±SD, ^#^ Median (range), ^§^ Median (95% Confidence interval), NA Not Available. CR complete response, PR partial resoponse, SD stable disease, PD progressive disease, cTACE Conventional transarterial chemoembolization, DEB-TACE Drug-eluting beads, TARE Transarterial radioembolization
