# Supplementary material for: Transarterial strategies for the treatment of unresectable hepatocellular carcinoma: A systematic review
Source: PLoS One. 2020 Feb 19;15(2):e0227475. doi: 10.1371/journal.pone.0227475 (PMC7029952; doi:10.1371/journal.pone.0227475)
Supplement: S3 Table — (DOCX) [file pone.0227475.s006.docx]

S3 Table: The comparison of main adverse events in the transarterial therapies for hepatocellular carcinoma

| Main adverse events | DEB-TACE vs. cTACE | TARE vs. cTACE | DEB-TACE vs. TARE |
| --- | --- | --- | --- |
|  | OR (95%CI); p value | OR (95%CI); p value | OR (95%CI); p value |
| Nausea /Vomiting | 0.25 (0.05- 1.21); 0.085 | 0.35 (0.07- 1.73); 0.199 | 1.03 (0.42- 2.50); 0.949 |
| Pain | 0.54 (0.07-4.05); 0.551 | 0.14 (0.01-2.51); 0.183 | 2.12 (0.30-15.03); 0.452 |
| Fatigue | 9.00 (3.99-20.31); 0.000 | 1.63 (0.25-10.59); 0.61 | NA |
| Infection/Fever | 0.45 (0.23-0.91); 0.027 | NA | 2.08 (0.11-38.4); 0.623 |
| Liver failure | 0.67 (0.36-1.23); 0.192 | 1.09 (0.47-2.49); 0.845 | 0.69 (0.18-2.61); 0.584 |
| Gastrointestinal bleeding | NA | 1.12 (0.29-4.38); 0.875 | 0.60 (0.09-3.99); 0.595 |

NA Not Available, OR Odds risk, cTACE Conventional transarterial chemoembolization, DEB-TACE Drug-eluting beads, TARE Transarterial radioembolization
