# Supplementary material for: Transarterial strategies for the treatment of unresectable hepatocellular carcinoma: A systematic review
Source: PLoS One. 2020 Feb 19;15(2):e0227475. doi: 10.1371/journal.pone.0227475 (PMC7029952; doi:10.1371/journal.pone.0227475)
Supplement: S4 Table — (DOCX) [file pone.0227475.s007.docx]

S4 Table: Summary of graded adverse events of transarterial therapies for hepatocellular carcinoma

| First author | Year | Treatment | Number(n) | Classification system | Adverse events（n） | | | | |
| --- | --- | --- | --- | --- | --- | --- | --- | --- | --- |
|  |  |  |  |  | Grade1 | Grade2 | Grade3 | Grade4 | Grade5 |
| McDevitt | 2017 | DEB-TACE-TACE | 26 | NCI-CTCAE | 68 | NA | 15 |  | NA |
|  |  | TARE | 24 |  | 47 | NA | 13 |  | NA |
| Kooby | 2010 | TARE | 27 | NCI-CTCAE | 3 | 1 | 3 | 3 | 2 |
|  |  | cTACE | 44 |  | 8 | 7 | 5 | 9 | 2 |
| Kolligs | 2015 | TARE | 13 | NCI-CTCAE | Grade 1 to 2, 1 | | Grade 3 to 4, 3 | | NA |
|  |  | cTACE | 15 |  | Grade 1 to 2, 3 | | Grade 3 to 4, 2 | | NA |
| Dhanasekaran | 2010 | DEB-TACE | 45 | NCI-CTCAE | NA | MA | 0 | 0 | 3 |
|  |  | cTACE | 26 |  | NA | NA | 0 | 0 | 2 |
| Lammer | 2010 | DEB-TACE | 93 | SWOG | 8 | 3 | 1 | 0 | NA |
|  |  | cTACE | 108 |  | 18 | 15 | 5 | 2 | NA |
| Megias | 2015 | DEB-TACE | 30 | NCI-CTCAE | NA | Grade 2 to 4, 31 | | | NA |
|  |  | cTACE | 30 |  | NA | Grade 2 to 4, 54 | | | NA |
| Philipp | 2011 | DEB-TACE | 22 | SIR | Grade 1 to 2, 10 | | Grade 3 to 4, 6 | | NA |
|  |  | cTACE | 22 |  | Grade 1 to 2, 7 | | Grade 3 to 4, 1 | | NA |
| Golfieri | 2014 | DEB-TACE | 89 | NCI-CTCAE | 26 | 9 | 6 | NA | 0 |
|  |  | cTACE | 88 |  | 42 | 38 | 20 | NA | 1 |

SWOG Southwestern Oncology Group; NCI-CTCAE National Cancer Institute Common Terminology Criteria for Adverse Events; SIR Society of Interventional Radiology clinical practice guidelines, NA Not Available, cTACE Conventional transarterial chemoembolization, DEB-TACE Drug-eluting beads, TARE Transarterial radioembolization
