# Supplementary material for: Transarterial strategies for the treatment of unresectable hepatocellular carcinoma: A systematic review
Source: PLoS One. 2020 Feb 19;15(2):e0227475. doi: 10.1371/journal.pone.0227475 (PMC7029952; doi:10.1371/journal.pone.0227475)
Supplement: S5 Table — (DOCX) [file pone.0227475.s008.docx]

S5 Table: Meta-regression analysis for overall survival

| Treatment | Variables | Coefficient (SE) | 95% CI | P-value for covariates | Heterogeneity τ2 | Proportion of between-study variance explained† |
| --- | --- | --- | --- | --- | --- | --- |
| DEB-TACE vs. cTACE | Study design | 1.16 (0.57) | 0.39-3.48 | 0.773 | 0 | 0 |
|  | Virus infection | 0.86 (0.49) | 0.24-3.08 | 0.79 | 0 | 0 |
|  | BCLC stage | 0.89 (0.69) | 0.15-5.36 | 0.89 | 0 | 0 |
|  | Proportion of Child-pugh A | 1.45 (2.45) | 0.03-77.81 | 0.83 | 0 | 0 |
|  | Treatment sessions | 0.37 (.39) | 0.03-5.58 | 0.39 | 0 | 0 |
| TARE vs. cTACE | Study design | 0.79 (0.26) | 0.32-1.97 | 0.522 | 0 | 0 |
|  | Virus infection | 0.60 (0.66) | 0.03-12.98 | 0.665 | 0 | 0 |
|  | Proportion of Child-pugh A | 1.01 (1.13) | 0.03-35.29 | 0.994 | 0 | 0 |

SE standard error; CI confidence interval; †Adjusted R^2^ for covariates, NA Not Available, cTACE Conventional transarterial chemoembolization, DEB-TACE Drug-eluting beads
