# Supplementary material for: Transarterial strategies for the treatment of unresectable hepatocellular carcinoma: A systematic review
Source: PLoS One. 2020 Feb 19;15(2):e0227475. doi: 10.1371/journal.pone.0227475 (PMC7029952; doi:10.1371/journal.pone.0227475)
Supplement: S6 Table — (DOCX) [file pone.0227475.s009.docx]

S6 Table: Key meta studies for TARE, DEB-TACE, and cTACE in the treatment of unresectable liver cancer

| First Author | Study Types | Comparsion | Included studies (n; RCT, Non-RCT) | Patients (n) | Effect Sizes | Overall Survival (Z, (95%CI, p); Heterogeneity, I2, P) | | |
| --- | --- | --- | --- | --- | --- | --- | --- | --- |
|  |  |  |  |  |  | 1 year | 2 year | 3 year |
| Ni 2014 | Meta | DEB-TACE/TARE vs. cTACE | 13 (2, 11) | 1834 (1233, 601) | OR | 1.38, (1.05-1.82, p=0.02); I2=39%, P=0.13 | 2.88, (1.18-7.05, P= 0.02); I2=58%, P=0.09 | 2.15, (1.18-3.91, p= 0.01); I2=0%, P =0.41 |
| Facciorusso 2016 | Meta | DEB-TACE vs. cTACE | 12 (4, 8) | 1449 (760, 689 ) | OR | 0.76, (0.48-1.21, p=0.25); I2=52%, P=0.03 | 0.68, (0.42-1.12, p=0.13); I2=69%, P＜0.001 | 0.57, (0.32-1.01, p=0.06); I2=65%, P=0.01 |
| Zou 2016 | Meta | DEB-TACE vs. cTACE | 9 (5, 4) | 868 (424 , 442) | OR | 1.41, (1.01-1.98); I2=96.6%, P＜0.001* | | |
| Chen 2017 | Meta | DEB-TACE vs. cTACE | 16 (4, 12) | 1832 (822 , 1010 ) | RR | 1.12, (1.03-1.23, P=0.007); I2=29%, P=0.19 | 1.26, (1.03-1.54, P=0.02); I2=58%, P=0.01 | 1.69 (1.02-2.81, p=0.04); I2=82%, P<0.0001 |
| Ludwig 2017 | Network Meta | DEB-TACE vs. TARE | 14 (3, 11) | DEB-TACE vs. cTACE 660 (331, 329); TARE vs. cTACE 1405 ( 405, 1, 000) | OR | 0.57, ( 0.36-0.92, p=0.02); I2=0 %, P > 0.5 | 0.65, (0.29-1.44, p=0.29) ; I2=71.3 %, P=0.002 | 0.71, (0.21-2.55, p=0.62); I^2^=79.8 %, P=0.002 |
| Casadei 2018 | Meta | TARE vs. DEB-TACE (cTACE) | 3 (3, 0) | 97 (49, 48) | OR | 1.31, (0.56-3.04, P=0.53); I2=0%, P=0.67 | NA | NA |
| Zhang 2015 | Meta | TARE vs. DEB-TACE/cTACE | 8 (0, 8) | 1, 499 ( 451, 1048) | HR | 0.74, (0.61-0.90, p=0.002); I2=38%, P=0.20* | | |
| Lobo 2016 | Meta | TARE vs. TACE | 5 (0, 5) | 553 ( 269, 284; ) | RR | 0.93, (0.81-1.08, p=0.33); I2=0%, P=0.8439 | 1.36, (1.05-1.76, p=0.02); I2=27.6%, P=0.2375 | 1.27, (0.88-1.84, p=0.20); I2=0%, P=0.7027 |
| Yang 2018 | Meta | TARE vs. cTACE | 11 (2, 9) | 1, 652 (528, 1124) | OR | 0.939, (0.705-1.251, p=0.66); I2=15.2%, P=0.303 | 0.57, (0.34-0.98, p=0.04); I2=55.7%, P=0.021 | NA |

**continued**

| First Author | Effect Sizes | PFS (Z, (95%CI, p); Heterogeneity, I2, P) | Response rate | | | | Safty | Conclusion |
| --- | --- | --- | --- | --- | --- | --- | --- | --- |
|  |  |  | Complete Response (CR) | Partial response (PR) | Stable disease (SD) | Progressive disease (PD) |  |  |
| Ni 2014 | OR | NA | 2.19, (1.31-3.64, p=0.003); I2=40%, P=0.09 | 0.73, (0.47-1.15, p=0.17); I2=46%, P=0.08 | 1.07, (0.79-1.44, p=0.67); I2=44%, P=0.11 | 2.15, (1.18-3.91, p=0.01); I2=71%, P=0.002 | 0.68, (0.46-1.00, P=0.05); I=0%, P=0.50* | Y90 or DEB is superior to cTACE in survival and treatment response |
| Facciorusso 2016 | OR | NA | 1.21 (0.69-2.12, p=0.51); I2=22%, P=0.25# |  |  |  | 0.85, (0.60-1.20, p=0.36); I2=5%, P=0.40* | DEB is not superior to cTACE |
| Zou 2016 | OR | NA | 1.38, (1.01-1.89); I2=0%, P=0.459 | 1.00, (0.67-1.49); I2=0%, P=0.834 | 1.21, (0.94-1.56); I2=0%, P=0.873# | 1.14, (0.81-1.58); I2=0%, P=0.643$ | 0.59, (0.41-0.84); I2=73.5%, P=0.01* | DEB is superior to cTACE |
| Chen 2017 | RR |  | 1.09 (0.94-1.25, P=0.25); I2=67%, P=0.0008# | 1.09, (0.98-1.20, p=0.11); I2=67%, P=0.0007* |  |  | Postembolization syndrome 0.87 (0.71-1.07, P=0.19) ; I2=10%, P=0.35; liver dysfunction 0.91 (0.25-3.23, P=0.88); I2=0%, P=0.52 | DEB is superior to cTACE in survival |
| Ludwig 2017 | OR | NA | 0.71, (0.13- 4.08, p=0.07); I2=79.1 %; P=0.03# |  |  |  | NA | DEB is superior to TARE in survival and treatment response |
| Casadei 2018 | OR | 0.23, (0.02-2.45, p=0.22); I2=76%, P=0.02 | 1.80, ( 0.51-6.30, p=0.36) ; I2=0%, P=0.51# |  |  | 0.61, (0.14-2.70, p=0.51); I2=0%, P=0.61 | NA | TARE has a similar outcomes TACE in survival and treatment response |
| Zhang 2015 | HR | 0.61, (0.41-0.89, p=0.01); I2=0%, P=0.74 | 1.92, (0.68-5.41); I2=0%, P=0.57 | 1.44, (1.02-2.04); I2=34%, P=0.20 | 1.05, (0.79-1.40); I2=0%, P=0.93 | 0.62 (0.37-1.04); I2=29%, P=0.23 | NA | TARE is superior to TACE (cTACE+DEB-TACE) in complication and survival |
| Lobo 2016 | RR | NA | 2.35, (0.76-7.28, p=0.14); NA | 0.85, (0.55-1.31, p=0.45); NA | 0.96, (0.38-2.42, p=0.92); NA | 1.07, ( 0.58-1.97, p=0.84) ; I2=71.8 %, P=0.029 | Pain 0.51, (0.36-0.72, p ＜0.01); fatigue ( 1.68, (1.08-2.62, p ＜0.01); Nausea and vomiting ( 0.83, (0.60-1.22, p=0.35), fever ( 1.16, (0.07-18.6, p=0.92), and other complications 1.09, (0.67-1.76, p=0.74) | TARE is superior to TACE in complication and survival |
| Yang 2018 | OR | NA | 0.78 ( 0.45-1.34, p=0.37); I2 =68.0%, P=0.002# |  |  |  | 1.48 (0.86, 2.53); I2=70.9%, P=0.002* | TARE is superior to TACE in complication and survival |

#Objective Response (OR)=CR+PR; $Disase Control (DC)=CR+PR+SD; * Total effect size; PFS: Progression-free survival
