# Supplementary material for: Transarterial strategies for the treatment of unresectable hepatocellular carcinoma: A systematic review
Source: PLoS One. 2020 Feb 19;15(2):e0227475. doi: 10.1371/journal.pone.0227475 (PMC7029952; doi:10.1371/journal.pone.0227475)
Supplement: S7 Table — (DOCX) [file pone.0227475.s010.docx]

S7 Table: Key limitations for comparison of radioembolisation and DEB-TACE in the treatment of unresectable liver cancer

| First Author | Limitations |
| --- | --- |
| Pitton 2015 | 1, This study has a very small populations (DEB-TACE vs. TARE, 12 vs. 12 ); 2, Baseline differences in TARE vs. DEB-TACE: alcohol/HCV/HBV/cryptogen (5/5/0/2 vs. 5/4/1/3) and AFP (3308 ± 10204 vs. 164 ± 529 ng/ml), and three or more tumor lesions (69% vs. 69%). |
| McDevitt 2017 | 1, This is a retrospective study; 2, Somall populations(DEB-TACE vs. TARE, 26 vs. 24 ) |
| Akinwande 2016 | 1, A retrospective study; 2, Baseline difference(TARE vs. DEB-TACE): Age (66.5 vs. 61.5), Karnofsky score (≥90%, 5% vs. 94%); 3, Sub-stratification was not performed in this study |
| Lance 2011 | 1, A retrospective study; 2, Different treatment modalities and regimens were employed for the chemoembolization and radioembolization regimens, including use of different particles in the radioembolization group and varying chemotherapy regimens in the chemoembolization group; 3, tumor response data is not unavailable. |
