# Supplementary material for: Transarterial strategies for the treatment of unresectable hepatocellular carcinoma: A systematic review
Source: PLoS One. 2020 Feb 19;15(2):e0227475. doi: 10.1371/journal.pone.0227475 (PMC7029952; doi:10.1371/journal.pone.0227475)
Supplement: S2 File — (DOCX) [file pone.0227475.s012.docx]

(((((((((((Yttrium-90) OR TheraSphere) OR SIR-spheres) OR radioembolisation) OR Y-90)) AND (((((((Drug eluting bead) OR DC Bead) OR Drug-eluting bead) OR QuadraSphere) OR CalliSpheres) OR HepaSphere) OR DC-Bead))) OR ((((((("Chemoembolization, Therapeutic"[Mesh]) OR transarterial chemoembolization) OR transcatheter arterial chemoembolization)) AND "Chemoembolization, Therapeutic"[Mesh])) AND (((((((Drug eluting bead) OR DC Bead) OR Drug-eluting bead) OR QuadraSphere) OR CalliSpheres) OR HepaSphere) OR DC-Bead))) OR ((((("Chemoembolization, Therapeutic"[Mesh]) OR transarterial chemoembolization) OR transcatheter arterial chemoembolization)) AND (((((Yttrium-90) OR TheraSphere) OR SIR-spheres) OR radioembolisation) OR Y-90)))) AND "Liver Neoplasms"[Mesh]
